# ASSOCIATION BETWEEN BLOOD PRESSURE AND COGNITIVE FUNCTION OF COMMUNITY-DWELLING OLDEST OLD IN OKINAWA, JAPAN

**DOI:** 10.1093/geroni/igz038.2391

**Published:** 2019-11-08

**Authors:** Yukihiro Namihira, Takashi Tokashiki, Akio Ishida, Yusuke Ohya, Hiroko H Dodge

**Affiliations:** 1 Department of Cardiovascular Medicine, Nephrology and Neurology, Graduate School of Medicine, University of the Ryukyus, Nakagami county, Okinawa, Japan; 2 Department of Neurology, National Hospital Organization Okinawa Hospital., Okinawa, Japan, Japan; 3 Department of Cardiovascular Medicine, Nephrology and Neurology, Graduate School of Medicine, University of the Ryukyus, Okinawa, Japan, Japan; 4 Department of Cardiovascular Medicine, Nephrology and Neurology, Graduate School of Medicine, University of the Ryukyus, Okinawa, Japan; 5 University of Michigan, Ann Arbor, Michigan, United States

## Abstract

Background: Adults 80 years and older are the fastest-growing segment of the Japanese population and face a high risk of cognitive decline. There are some evidences connecting hypertension to cognitive decline. In mid-life hypertension is known to have influence the cognitive decline in older age. However, a few study have examined the association between hypertension or vascular stiffness and cognitive function among elderly over 80 years old. We analyzed the associations between vascular stiffness and cognitive function among relatively healthy community-dwelling non-demented oldest old. Method: Data came from the Keys to Optimal Cognitive Aging (KOCOA) study; an ongoing cohort of relatively healthy volunteers aged over 80 years old, living in Okinawa, Japan. In 2017, 105 non-demented (Clinical Dementia Rating < 1) subjects completed three kinds of examination for vascular function (75 % female, mean age (SD) 84.0 (3.0)). We categorized subjects into low and high cognitive function groups using Montreal Cognitive Assessment (MoCA) (25/26 as a cutpoint). Logistic regression models were used to examine the association between cognitive and vascular functions. Results: Narrower pulse pressure, an indicator of lower arterial stiffness, was associated with better cognitive function among subjects, after adjusting for gender, age, and education (p≦0.05), although systolic and diastolic blood pressure were not. Conclusion: Our findings suggest that narrower pulse pressure is related with cognitive preservation. The present study supports the hypothesis that lower arterial stiffness is related with better cognitive function even among the oldest old.

